# Cook County Hospital

**Published:** 1877-04

**Authors:** J. H. Wm. Meyer

**Affiliations:** House Physician


### Clinical Reports.

### COOK COUNTY HOSPITAL.

### MEDICAL DEPARTMENT.

Cases Reported by J. H. Wm. Meyer, M. D., (House Physician).

*Phthisis Pulmonum, Erysipelas, Meningitis.*

H. Anderson, a Norwegian, cabinet-maker, aged 62, was admitted to the hospital, Jan. 26, 1877. He had long suffered from cough, and pain in his chest; expectoration of a thick, tenacious sputum, at first white, subsequently yellow; emaciation, night-sweats, and other features of consumption.

Physical examination revealed depression below right clavicle; diminished respiratory movement of that side; dullness, broncho-vesicular breathing, bronchophony, and increased vocal fremitus over the upper lobe of right lung. Appropriate treatment was ordered.

On Feb. 8th, there was apparent a red, glazed swelling of the face, presenting several blebs; tenderness to the touch; patient had been delirious the previous night. He was directed to take a saline laxative, and a powder containing quinine and opium; also to make a local application of camphorated oil. In the evening he was rational, but became delirious again during the night. On the following morning (Feb. 9), he was ordered the tinct. ferri chloridi, in half drachm doses, four times daily.

On the 11th, the redness was evidently fading; ung. zinci oxidi was substituted for oleum camph.

By the 13th, desquamation was well established over the entire face, but patient was almost constantly delirious.

Death occurred at 5 A. M., Feb. 15th.

An examination was made six hours after death.

On penetrating the skull and dura mater, about two ounces of serum escaped; the arachnoid was found to be covered with lymph; the pia mater injected; between the convolutions were shreds of lymph. The upper lobe of the right, and the lower lobe of the left lung were infiltrated with yellow, caseous matter.

*Phthisis and Bright's Disease.*

Ph. Conway, aged 27 years, cooper, was admitted, Nov. 15, 1876.

General health of patient has been good, till about three years ago. As far as known, there is no hereditary predisposition to consumption. Has not been in the habit of drinking to excess.

Three years ago last spring, he took cold, and had severe pain in the chest, extending through the shoulders. Six months later, he had a hemorrhage from the lungs, losing considerable blood. Expectorated blood, and coughed a great deal, at varied intervals, all that winter. He would expectorate about half a teacupful of blood at a time, but at one time lost about a pint. He steadily declined in health, till about two years ago, he was obliged to give up work. Appetite has been very poor; bowels generally costive. Had night-sweats. Three months ago, patient noticed that he was making more than the normal quantity of urine, and the amount has increased up to the present time. He now voids during the night about a gallon of porter-colored urine. Has had for the last three months a heavy pain in the region of the kidneys. Drinks a great amount of water. Last June, his feet began to swell; if out of bed, the swelling would be greatest in the evening; while in the morning, the feet were smaller again.

No tumefaction has ever appeared in any other region.

On admission, patient very much emaciated; voice hoarse; face sallow. There is an anæmic aspect of the skin, and visible mucous membranes. The tongue is tremulous, and coated whitish. Appetite poor, but has great thirst; bowels costive, but has a frequent desire to urinate, especially at night.

Complains of pain in chest and back. Feet pit on pressure.

Examination of chest: over left upper region anteriorly, dullness on percussion with broncho-vesicular respiration, bronchophony, depression below the clavicle, and diminished respiratory movements on that side; some mucous râles.

Examination of urine shows S. G. 1003. Heat and nitric acid give deposit of albumen. Ordered ferri et quiniæ cit. gr. v four times a day, and cod-liver oil after meals.

Nov. 25th—Patient sits up in bed most of the time; he cannot breathe in the recumbent posture.

Dec. 15th—Complains of some diarrhœa. Ordered milk diet.

Dec. 25th—Vomits some; bowels are quite loose. He has no œdema, but is very thin.

Feb. 5th—Urine slightly acid, pale yellow, and viscid. After standing, a light, whitish, flocculent precipitate falls; copious deposit by heat and nitric acid.

Feb. 6th—Profuse diarrhœa. Ten passages last twelve hours.

℞ Plumbi acetatis.................. gr. i.
Pulveris opii................... gr. j.

every four hours.

Feb. 13th—Died.

Feb. 16th—Could not make a complete post mortem, but managed to get the kidneys, which were small, smooth, weighing 8½℥—one, 4⅛ʒ; the other, 4⅜ʒ. Capsule firmly adherent; on section, thickness of cortical substance diminished.

### SURGICAL DEPARTMENT.

(Reporter C. F. Fenn, M. D.)

### *Urinary Fistula Opening in Scarpa's Triangle.*

In the Surgical Clinic of the 14th March, Prof. Bogue presented an unusual case, on which he performed the operation of cystotomy for the cure of a urinary fistula, which had its external opening on the inner aspect of the thigh. The history of the case is briefly this: A man, 55 years of age, having a light occupation and previously good health, began to have more or less pain within the pelvis on the right side. Eventually there was a considerable discharge of pus from the bladder.

After several weeks of disquiet of this kind, a small tumor suddenly appeared in the right groin. Its development was from beneath Poupart's ligament, like that of a psoas abscess. It continued to grow and to descend until it reached the apex of Scarpa's triangle. When first examined, two months ago, there was a smooth fluctuating tumor four inches in diameter at the base, and an inch and a half in elevation. Its appearance was precisely that of a cold abscess, the surface having become red and inflamed from friction or from the uniform weakening of its walls. That this was a collection of pus from a distant source, seemed to be the only conclusion possible. When the tumor was lanced, urine, instead of pus, came forth. The walls of the cavity collapsed, and the punctured opening remained as the outlet of an urinary fistula. Observing the patient after this, it was found that the act of evacuating the bladder in the usual way occasioned also a flow of urine through the fistula. If the bladder became filled at any time, the flow by the fistula was involuntary: but as long as any considerable accumulation of urine was prevented by frequently voiding the bladder, the flow of urine by the abnormal channel was limited to the time of micturition.

The operation of cystotomy was performed in order to allow the valvular opening or diverticulum to heal that must have been produced in the course of previous abscesses within the peri-vesical cellular tissue.

The operation resulted in the discovery and removal of a smooth ovoid calculus, weighing about one drachm, which was imbedded in an ulcerated cavity in the prostate gland.

